# Risk factors associated with malaria infection along China–Myanmar border: a case–control study

**DOI:** 10.1186/s12936-022-04312-5

**Published:** 2022-10-09

**Authors:** Jian-Wei Xu, Dao-Wei Deng, Chun Wei, Xing-Wu Zhou, Jian-Xiong Li

**Affiliations:** Yunnan Institute of Parasitic Diseases, Yunnan Provincial Centre of Malaria Research; Yunnan Provincial Key Laboratory of Vector-borne Disease Control and Research; Yunnan Institute of Parasitic Diseases Innovative Team of Key Techniques for Vector Borne Disease Control and Prevention; Training Base of International Scientific Exchange and Education in Tropical Diseases for South and Southeast Asia, Puer, 665000 China

**Keywords:** Malaria, Risk factors, Cross-border collaboration, China, Myanmar, Greater Mekong Subregion

## Abstract

**Background:**

The World Health Organization (WHO) has certificated China malaria free, but imported malaria is a continuous challenge in preventing reintroduction of malaria in the border area of China. Understanding risk factors of malaria along China–Myanmar border is benefit for preventing reintroduction of malaria in China and achieving the WHO’s malaria elimination goal in the Greater Mekong Subregion (GMS).

**Methods:**

This is a case–control study with one malaria case matched to two controls, in which cases were microscopy-confirmed malaria patients and controls were feverish people with microscopy-excluded malaria. A matched logistic regression analysis (LRA) was used to identify risk factors associated with malaria infection.

**Results:**

From May 2016 through October 2017, the study recruited 223 malaria cases (152 in China and 71 in Myanmar) and 446 controls (304 in China and 142 in Myanmar). All the 152 cases recruited in China were imported malaria. Independent factors associated with malaria infection were overnight out of home in one month prior to attendance of health facilities **(**adjusted odd ratio [AOR] 13.37, 95% confidence interval [CI]: 6.32–28.28, P < 0.0001), staying overnight in rural lowland and foothill **(**AOR 2.73, 95% CI: 1.45–5.14, P = 0.0019), staying overnight at altitude < 500 m **(**AOR 5.66, 95% CI: 3.01–10.71, P < 0.0001) and streamlets ≤ 100 m **(**AOR9.98, 95% CI: 4.96–20.09, P < 0.0001) in the border areas of Myanmar; and people lacking of knowledge of malaria transmission **(**AOR 2.17, 95% CI: 1.42–3.32, P = 0.0004).

**Conclusions:**

Malaria transmission is highly focalized in lowland and foothill in the border areas of Myanmar. The risk factors associated with malaria infection are overnight staying out of home, at low altitude areas, proximity to streamlets and lack of knowledge of malaria transmission. To prevent reintroduction of malaria transmission in China and achieve the WHO goal of malaria elimination in the GMS, cross-border collaboration is continuously necessary, and health education is sorely needed for people in China to maintain their malaria knowledge and vigilance, and in Myanmar to improve their ability of personal protection.

**Supplementary Information:**

The online version contains supplementary material available at 10.1186/s12936-022-04312-5.

## Background

Malaria is an anopheline mosquito-borne parasitic disease [1], and remains one of main global public health threats. Due to health service disruptions during the coronavirus disease 2019 (COVID-19) pandemic, there were an estimated 241 million malaria cases in 2020, increased from 227 million in 2019, and malaria deaths increased by 12% compared with 2019, to an estimated 627 thousand [2]. The goal of the World Health Organization (WHO) is to eliminate malaria in all countries of the Greater Mekong Subregion (GMS) by 2030 [3]. Cross-border malaria transmission keeps a regional impediment towards malaria elimination in the GMS [4]. China (Yunnan) has malaria ecology and vector system similar to those of five other countries in the GMS. The investigation of risk factors of border malaria can provide scientific evidences in planning malaria elimination in the GMS. After the national malaria programme and its partners dedicated efforts for seven decades, the WHO certified China malaria-free status on June 30, 2021 [5]. However, Yunnan Province shares 4060 km of border with Myanmar (1997 km), Laos (710 km) and Vietnam (1353 km). The interruption of malaria transmission in northern Vietnam has been reported [6, 7]. Malaria towards the subnational elimination goal has also reported in northern Laos [8]. Threat of imported malaria is therefore slight from Vietnam and Lao PDR to China [9]. Malaria was also effectively controlled in most parts of the China–Myanmar border [10]. However, Yunnan borders Myanmar’s five local ethnic special regions that are partly out of the central government management of Myanmar Union. Some parts of the special regions, such as Kachin Special Region II (KR2) and Kongkang Autonomous Regions, are currently in military conflicts. Health services of the Ethnic Health Organizations are too weak to control malaria effectively. Malaria is highly endemic in some of the Myanmar border area [11]. Knowing risk factors of malaria infection well would be helpful in preventing reintroduction of malaria transmission in Yunnan and elimination in Myanmar.

In high endemic situation, risk factors can be investigated through representative cross sectional surveys. In malaria elimination setting, malaria is increasingly imported, caused by *Plasmodium vivax*, and clustered demographically in adult men with shared epidemiological risk factors [12]. In the situation of low incidence and elimination, the sectional survey is unlikely to adequately identify malaria cases and associated risk factors because the sectional surveys are unlikely to adequately detect malaria [13]. Case–control study is a well-established tool to investigate rare diseases and identify associated demographic, behavioural, and clinical risk factors, being particularly appropriate for rare diseases. As yet, this method has not been extensively used to the epidemiological study of malaria, which has mostly been investigated in high-endemic settings [14]. To further understand risk factors of malaria infection along the China–Myanmar border, a case–control study was conducted to investigate factors associated malaria infection in five prefectures of China (Yunnan) and a hospital in the KR2 of Myanmar.

## Methods

### Study design and sample size

The study was a case–control design with one malaria case matched to two controls. The malaria cases were diagnosed by microscopy and the controls were excluded malaria by microscopy too. They were febrile patients who attended the same health facility within a week. To mitigate the potential confounding of age, sex and health status, the controls were also matched on the basis of age (± 5 years), gender and health status without complicated diseases. The intended sample size was calculated by using a 95% two-sided confidence level, 80% power, 20% of cases with exposure and 10% of controls with exposure in Epi Info 7.0 (Centers for Disease Control and Prevention, USA). The sample size calculated was at least a total of 158 cases and 316 controls.

### Study sites

**Fig. 1:**
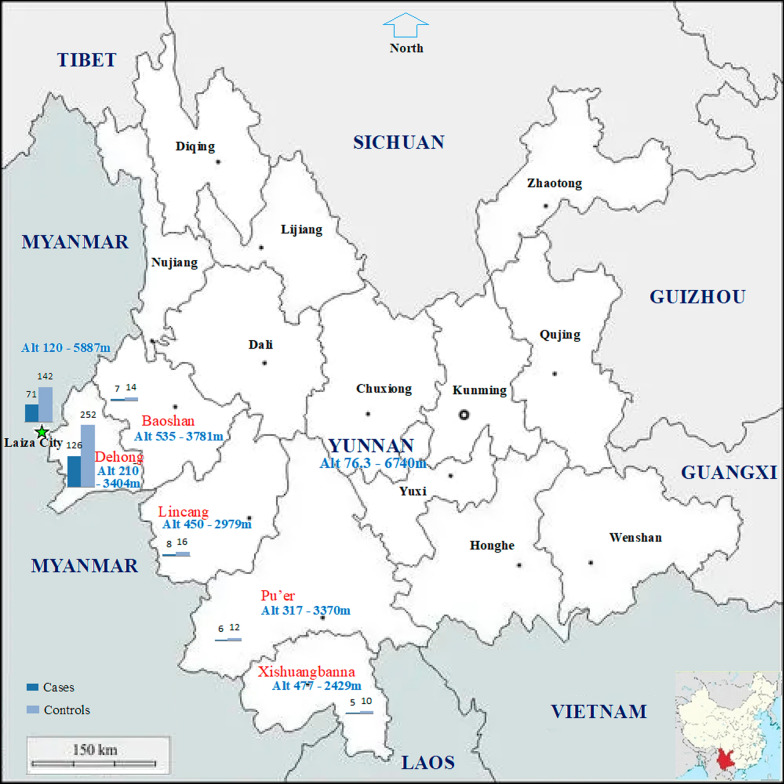
Study site locations, altitudes and number of subjects enrolled at each site

More than 95% of the study area is mountainous. The altitudes are from 76.4 m to 6740 m in Yunnan, China and from 120 to 5887 m in Kachin State, Myanmar. In order to recruit enough number of malaria cases for this study, the study covered border areas of five prefectures, namely, Dehong, Baoshan, Lincang, Pu'er and Xishuangbanna in China and the KR2 based on the epidemiology of malaria [10, 11, 14–26] (Fig. 1). The Laiza City and nearby areas in the KR2 of Myanmar is the hottest spot of malaria along the China–Myanmar border [11]. More than 80% of malaria cases in Yunnan in recent five years were imported from the Laiza City and nearby areas. In recent five years, Yingjiang County of Dehong Prefecture neighbouring the Laiza City and nearby areas reported more than one third of imported malaria cases in Yunnan [25, 26]. The subjects of this study were enrolled from 37 health facilities in five border prefectures of China and the Laiza City Hospital in Myanmar (Fig. 1).

Microscopy for malaria parasites is well performed in all of the 38 health facilities involved. Study sites selected in China were former hyperendemic areas where are at a high risk of reestablishing malaria transmission due to existing vectors. The study area with hot climate and adequate precipitation is suitable for the growth and reproduction of mosquitoes and the transmission of malaria. With a complex vector community, *Anopheles dirus* and *Anopheles minimus* have been identified as two primary vectors [18, 20]. *An. dirus* is a well-known exophagic vector, and *An. minimus* is mainly endophagic with increasing exophagic due to frequent use of insecticides. Perennial malaria transmission can occur in the study area. The peak season of malaria transmission is from June through August each year [18, 20].

### Laboratory diagnosis and structure interview

Microscopy for malaria parasites is one of normal test items in the enrolled health facilities. Laboratory technicians were responsible for recruiting the cases and controls. A finger or earlobe prick in febrile patients was performed to generate blood smears. The febrile patients were ones with axillary temperatures of not lower than 37.3 °C within 48 h. The blood slides were stained with Giemsa for microscopy. The malaria cases were recruited from microscopy-confirmed malaria patients between May 2016 and October 2017. Meanwhile, the feverish people with microscopy-excluded malaria were enrolled as controls following informed consent obtained. When laboratory technicians were not excellent, they could not detect malaria from blood slides with low parasite densities, the malaria patients with low parasite densities might be excluded from the malaria case group, and then into the control group. When the laboratory technicians read a negative slide as positive one, the febrile patient might into the case group instead of into the control group. As the quality control, all blood slides were re-read by an expert microscopist with the WHO malaria microscopy level one certificate for the secondary confirmation, namely, to exclude subjects with false positivity and false negativity. A paper-based questionnaire was administered to the subjects in Mandarin in China’s health facilities and in Kachin ethnical language in the Laiza City Hospital of Myanmar. The questionnaire has 36 questions, covering demographics, occupation, activities, travel history, housing condition, local ecology, socio-economic status, behaviour, malaria awareness and knowledge, and preventive measures. In the questionnaire, details of principal household components were recorded to construct a family wealth index (FWI) (Table 1) [27–30].

**Table 1:** Principal components for construction of the family wealth index (FWI)

FWI	Housing characteristics	Transportation tools	Family belongings
1. Most poor	Bamboo walls and sheet iron roofs	None	None or chickens
2. Mid low	Wood walls and sheet iron roofs	Bicycles	Pigs or goats
3. Middle	Brick walls, wood girders and terracotta roofs	Motorcycles	Cattle or horses
4. Mid high	Brick concrete walls and terracotta roofs	Tractors	TV sets or refrigerators
5. Least poor	Steel and concrete	Cars	Shops or elephants

### Statistical analysis

Data were entered and cleaned in Excel 2007, and then were analyzed in Epi Info 7.2. The statistical associations were based on matched analyses. For the case and the control group, the frequency and its 95% confidence interval (CI) of each predictor variable were calculated, and then compared using a Fisher exact chi-square test. A multivariate logistic regression analysis (LRA) was used to identify risk factors associated with malaria infection. In modeling strategy, the independent variables of univariate LRA were items with values of P < 0.10 in the chi-square test between the cases and the controls. The independent variables were included in the multivariate LRA model if they had a value of p < 0.25 in the univariate LRA. Non-response answers were treated as missing value and therefore excluded from the analyses [14, 31]. The data of subjects recruited in China and Myanmar were analysed, respectively. And then the same methodology was used to analyse the data of overall subjects. The risk factors of malaria infection were finally determined by comparing across the results from the three analyses (for subjects enrolled in China and Myanmar, and the combined).

## Results

### Subjects’ characteristics

A total of 223 malaria cases and 446 matched controls were recruited to participate in the study. Based on the China Information System for Disease Control and Prevention (CISDCP), all 152 malaria cases enrolled in China satisfied with the CISDCP criteria and were categorized into imported malaria from Myanmar. The CISDCP criteria for an imported malaria case is the malaria patient 1) with overnight stay history in endemic areas of other countries within one month prior to malaria that is diagnosed by laboratory test and 2) with robust evidences of malaria free in the malaria patient’s community in China. A total of 304 matched controls were recruited in the same health facilities. Of the 152 cases, 141 (92.7%) were *Plasmodium vivax* malaria and 10 (6.6%) *Plasmodium falciparum* and 1 (0.7%) mixed infection of *P. vivax and P. falciparum;* the numbers of male, aged < 16, 16–59 and > 60 years old were 120 (79.0%), 10 (6.6%), 138 (90.8%) and 4 (2.6%), respectively (Additional file 1: Table S1)*.* A total of 71 cases and 142 matched controls were enrolled from the Laiza City Hospital in the KR2 of Myanmar. Of the 71 cases, 69 (97.2%) were *P. vivax* malaria, 1 (1.4%) *P. falciparum* and 1 (1.4%) mixed infection of *P. vivax and P. falciparum;* the numbers of male, aged < 16, 16–59 and > 60 years old were 43 (60.6%), 17 (24.0%), 53 (74.6%) and 1 (1.4%), respectively (Additional file 1: Table S2). Among overall 669 research subjects, 489 (73.1%) were male and 180 (26.9%) female; and mean age was 30.6 years old (median: 29.0, range: 1–76). Most of the 223 cases were young male adults, and the 446 controls were matched by age and gender. Of the 223 confirmed malaria case patients, 210 (94.2%) cases were *P. vivax* malaria, 11 (4.9%) *P. falciparum*, 2 (0.9%) mixed infection of *P. vivax and P. falciparum* (Additional file 1: Table S3). The geometric mean parasite density of the malaria patient cases was 3347.5 (rang 174–65 418) parasites per ul. Of the malaria cases, the numbers of male, aged < 16, 16–59 and > 60 years old were 163 (73.1%), 27 (12.1%), 191 (85.7%) and 5 (2.2%), respectively (Additional file 1: Table S3).

### Risk factors of malaria cases enrolled in China

The malaria case group and the control group were similar in terms of demographics, proportions living in towns, residence proximity to forest, vector control measures, knowledge of malaria prevention, and engagement in trading, road- building and mining, sight-seeing and lumbering activities in one month prior to enrolling into the study (P > 0.10) (Additional file 1: Table S1). The matched univariate and multivariate LRA identified five independent risk factors associated with malaria infection from subjects recruited in China. Two of the risk factors were overnight in Myanmar **(**adjusted odd ratio [AOR] 30.31, 95% confidence interval [CI]: 15.42–59.58, P < 0.0001) and overnight out of home (AOR 69.71, 95%CI: 8.59–261.47, P < 0.0001) within one month prior to enrolling into the study. Three of the risk factors were staying overnight in rural lowland and foothill (AOR 3.05, 95%CI: 1.59–5.84, P = 0.0008), staying overnight at altitude < 500 m (AOR 4.59, 95%CI: 2.35–8.97, P < 0.0001) and staying overnight nearby streamlets (≤ 100 m) (AOR 8.08, 95%CI: 2.23–29.25, P = 0.0015) for malaria cases in Myanmar within one month prior to attendance of health facilities (Table 2).

**Table 2:** Risk factors associated malaria infection in the Yunnan border area, China

Factors	OR (95%CI)	*P* value	AOR (95%CI)	*P* value
School education ≤ 6 years	1.72 (1.14–2.60)	0.0103	1.20 (0.55–2.61)	0.6476
Family wealth index < 3	2.56 (1.70–3.86)	< 0.0001	1.55 (0.70–3.45)	0.2798
Residence ecology in one month prior to attendance of health facilities				
Overnight in Myanmar	35.90 (20.53–62.79)	< 0.0001	30.31 (15.42–59.58)	< 0.0001
Overnight out of home	129.89 (58.91–286.36	< 0.0001	69.71 (8.59–261.47)	< 0.0001
Rural lowland and foothill	2.56 (1.69–3.87)	< 0.0001	3.05 (1.59–5.84)	0.0008
Altitude < 500 m	7.59 (4.89–11.77)	< 0.0001	4.59 (2.35–8.97)	< 0.0001
Water body ≤ 100 m	9.40 (4.61–19.17)	< 0.0001	1.22 (0.005–331.49)	0.9446
Streamlets ≤ 100 m	37.95 (15.94–90.37)	< 0.0001	8.08 (2.23–29.25)	0.0015
Proximity to shrub grass and forest	4.80 (3.14–7.35)	< 0.0001	1.53 (0.78–2.99)	0.2116
Wood and thatched houses or cottages	40.21 (16.86–95.88)	< 0.0001	2.20 (0.47–10.40)	0.3201
No screened windows and doors	8.77 (4.29–17.96)	< 0.0001	5.80 (0.31–110.16)	0.2419
No measures against mosquito bites	1.05 (0.68–1.64)	0.8208	-	-
Use of untreated nets	0.9465 (0.63–1.41)	0.5489	-	-
Working as day laborers	1.3261 (0.44–31)	0.5656	-	-
Building houses, visiting relatives and friends	4.49 (2.47–8.16)	< 0.0001	1.79 (0.80–3.98)	0.1553
Don’t know malaria symptoms	0.62 (0.40–0.97)	0.0347	0.62 (0.24–1.60)	0.3269
Don’t know malaria transmission	1.47 (0.95–2.27)	0.0829	1.69 (0.72–3.97)	0.2287
No consideration of malaria prevention prior to entering endemic areas	2.63 (1.57–4.39)	0.0002	10.02 (0.99–101.30)	0.0509
Non co-decision	5.10 (2.44–10.65)	< 0.0001	1.36 (0.02–99.57)	0.8894
Availability of foreigners nearby home	2.36 (1.51–3.69)	0.0002	2.98 (0.32–27.74)	0.3375

OR, odd ratio; CI, confidence interval; AOR, adjusted odd ratio

### Risk factors of malaria cases enrolled in Myanmar

The malaria case group and control group were similar in terms of demographics, FWI, nationality, country overnight in one month prior to enrolling into the study, proximity of vegetation, screens of windows and doors, types of bed nets used, knowledge of malaria prevention (P > 0.10) (Additional file 1: Table S2). The matched univariate and multivariate LRA identified four independent risk factors associated with malaria infection from subjects enrolled in Myanmar, namely, staying overnight out of home within one month prior to attendance of health facilities (AOR 2.72, 95%CI: 1.40–5.29, P = 0.0033), staying overnight in Laiza city (AOR 3.44, 95%CI: 1.55–7.64, P = 0.0023), staying overnight at altitude < 500 m (AOR 2.81, 95%CI: 1.21–6.51, P = 0.0161) and streamlets ≤ 100 m (AOR4.63, 95%CI: 1.04–20.57, P = 0.0441) (Table 3).The Laiza city is located in a mountain valley with an altitude from 230 to 260 m and nearby a river and a few of streamlets. Staying in the Laiza city and nearby areas is at a high risk of malaria infection.

**Table 3:** Risk factors associated malaria infection in Kachin Special region II, Myanmar

Factors	OR (95%CI)	*P* value	AOR (95%CI)	*P* value
Overnight out of home	3.37 (1.82–6.3)	0.0001	2.72 (1.40–5.29)	0.0033
Laiza city	2.95 (1.54–5.62)	0.0011	3.44 (1.55–7.64)	0.0023
Altitude < 500 m	4.1300 (1.96–8.71)	0.0002	2.81 (1.21–6.51)	0.0161
Streamlets ≤ 100 m	2.80 (1.35–5.83)	0.0058	4.63 (1.04–20.57)	0.0441
Wood, earth and thatched houses or cottages	0.33 (0.17–0.64)	0.0009	0.65 (0.30–1.41)	0.2753
Not using bed nets	0.36 (0.13–0.98)	0.05	0.93 (0.29–2.96)	0.8938
Don’t know malaria transmission	1.81 (0.94–3.45)	0.0744	1.44 (0.61–3.46)	0.4082
Don’t know nearby endemic areas	0.38 (0.20–0.75)	0.0053	0.56 (0.23–1.40)	0.2136
No consideration of malaria prevention	0.53 (0.30–0.94)	0.0429	0.97 (0.49–2.65)	0.9944
Senior member decision	1.143 (0.33–3.90)	0.8313	–	–

*OR* odd ratio, *CI* confidence interval, *AOR* adjusted odd ratio

### Risk factors of overall malaria cases

Among overall subjects enrolled in China and Myanmar, the malaria case group and the control group were similar in terms of demographics, education, knowledge and awareness in malaria prevention (P > 0.10) (Additional file 1: Table S3). The matched univariate and multivariate LRA identified six independent risk factors associated with malaria infection. Five of the risk factors were as same as identified from the subsample recruited in China, namely, staying overnight in Myanmar within one month prior to attendance of health facilities, staying overnight out of home, especially staying in rural lowland and foothill, staying overnight at low altitude and proximity (≤ 100 m) of streamlets (Table 4). Due to increased sample size, the variable of no knowledge of malaria transmission, namely, the malaria cases lacking knowledge of malaria transmission by mosquitoes compared to the controls, was identified as one of risk factors associated with malaria infection (AOR2.17, 95%CI: 1.42–3.32, P = 0.0004) (Table 4).

**Table 4:** Risk factors associated malaria infection along China-Myanmar border

Factors	OR (95%CI)	*P* value	AOR (95%CI)	*P* value
Family wealth index < 3	0.60 (0.33–1.06)	0.0856	0.43 (0.15–1.26)	0.1237
Non-Chinese	5.57 (2.37–13.08)	0.0001	3.01 (0.64–14.17)	0.1633
Residence ecology in one month prior to attendance of health facilities				
Overnight in Myanmar	20.75 (10.86–39.65)	< 0.0001	11.25 (5.32–23.79)	< 0.0001
Overnight out of home	18.02 (10.77–30.16)	< 0.0001	13.37 (6.32–28.28)	< 0.0001
Rural lowland and foothill	2.9485 (2.06–4.22)	< 0.0001	2.73 (1.45–5.14)	0.0019
Altitude < 500 m	12.05 (6.89–21.09)	< 0.0001	5.66 (3.01–10.71)	< 0.0001
Water body ≤ 100 m	10.95 (5.31–22.59)	< 0.0001	1.25 (0.08–18.34)	0.8737
Streamlets ≤ 100 m	10.5250 (5.78–19.16)	< 0.0001	9.98 (4.96–20.09)	< 0.0001
Proximity to shrub grass and forest	4.24 (2.74–6.56)	< 0.0001	2.70 (0.69–10.65)	0.1549
Wood and thatched houses or cottages	4.67 (3.22–6.77)	< 0.0001	0.53 (0.20–1.38)	0.1928
No screened windows and doors	4.51 (2.56–7.94)	< 0.0001	1.39 (0.26–7.49)	0.7023
No measures against mosquito bites	0.85 (0.55–1.30)	0.4481	–	–
Stable salary income	1.20 (0.74–1.91)	0.4658	–	–
Farming, building and visiting relatives and friends	3.07 (1.87–5.03)	< 0.0001	1.21 (0.51–2.83)	0.6657
Don’t know malaria symptoms	0.53 (0.35–0.81)	0.0030	0.47 (0.18–1.19)	0.1100
Don’t know malaria transmission	1.69 (1.17–2.45)	0.0054	2.17 (1.42–3.32)	0.0004
Don’t know nearby endemic areas	0.52 (0.34–0.79)	0.0019	0.64 (0.26–1.56)	0.3210
Senior member or husband decision	2.99 (1.76–5.08)	0.0001	1.10 (0.44–2.74)	0.8347
Availability of foreigners nearby home	2.95 (1.93–4.50)	< 0.0001	1.12 (0.46–2.75)	0.8048

*OR* odd ratio, *CI* confidence interval, *AOR* adjusted odd ratio

## Discussion

### Risk factors of malaria infection

The low altitude leads to hot temperature and adequate precipitation and then abundant breeding sites of anopheline mosquitoes in the China–Myanmar border area. All results of former investigations documented that people in low altitude areas were at a high risk of malaria infection. A retrospective case–control study suggested that independent risk factors associated with malaria infection were overnight in the lowland, foothill and half-hill areas, and near anopheline mosquito breeding sites in the China–Myanmar border area [14]. A cross-sectional study reported that independent risk factors for slide positivity were age, lower altitude, lack of knowledge about malaria transmission and symptoms, inaction against mosquito bites and delayed treatment-seeking in the Salween river valley of Shan Special Region II (SR2), northern Myanmar [16]. In this study, the comparison of multivariate LRA results between the subjects recruited in China and Myanmar indicated that the risk factors associated with malaria infection were mainly overnight out of homes within one month prior to illness, staying in rural lowland and foothill, staying at altitude < 500 m and streamlets ≤ 100 m in the border areas of Myanmar. For the subsample enrolled in China, the overnight in Myanmar was a part of overnight out of home. For the subsample enrolled in Myanmar, the Laiza city and nearby areas was altitude < 500 m and streamlets ≤ 100 m, staying in the Laiza city and nearby areas was therefore at a high risk of malaria infection. The LRA result of the overall sample indicated that no knowledge of malaria transmission was a risk factor of malaria infection due to the increased sample size. The proportion of case-patients with knowledge of malaria transmission (67.3%) was significantly lower than that of controls (77.6%) (P = 0.0055). This indicates that health education on malaria should be necessary for people in both China and Myanmar. The public health in China should maintain people’s malaria knowledge and vigilance, and remind people using personal protection against malaria infection when they are in the endemic areas of other countries. Cross-border workers should be educated on preventive measures for malaria through effective behaviour change communication [25].

### Malaria along China–Myanmar border and other parts in Southeast Asia

In most areas of the Southeast Asia, the year-round high rainfall and temperatures, and abundant malaria vectors lead to persistent and intense malaria transmission. Most parts of the GMS are low latitude and altitude, and suitable for fertility of malaria vectors and persistent malaria transmission [32]. Forests are traditionally considered as a major determinant of malaria risk in the GMS [33, 34]. In Vietnam a high proportion of malaria cases and deaths were reported in the central mountainous and forested areas [35]. In Myanmar, most of malaria cases were reported to occur in forest or forest fringe areas, and loggers and gem miners were at high risk of malaria [36, 37]. However, the China–Myanmar border areas with altitude from 200 m through 5887 m are mostly mountainous. The low altitude areas have high temperatures, adequate mosquito breeding sites and malaria vectors [14]. After malaria was effectively controlled along the China–Myanmar border [10], the major malaria hot spots are the Laiza and its nearby areas of the KR2 [18, 19], the Salween river valley of the SR2 [15, 16] and the small golden triangle at China–Myanmar–Laos border in the Mekong River Valley [4, 11] in the northern Myanmar. All the three malaria hot spots are low altitude. The two former investigations [14, 16] and this study did not identify malaria in association with forest in the northern Myanmar. This documents that the heterogeneity and complexity of malaria should be considered in planning control and elimination programmes in the GMS [38].

### Malaria epidemiological characteristics along China–Myanmar border

In eliminating settings, malaria cases are increasingly male, adult, clustered geographically, imported among migrant and other hard-to reach groups, and caused by *P. vivax* [12, 14]*.* All of 152 malaria cases recruited in China were imported among migrants, and most of them were male, adult and *P. vivax* malaria. In the chi-square test, the proportions of male and aged ≥ 16 year old malaria cases recruited in China were significantly higher than the proportions of malaria cases recruited in Myanmar (male, x^2^ = 7.4081, P = 0.0065; age, x^2^ = 12.1295, P = 0.0060). The proportions of *P. vivax* between two subsamples were not significant (x^2^ = 1.0111, P = 0.3146), which is one of evidences of the same infection locations. When the high percentage of *P. vivax* malaria indicates the success in control of *P. falciparum* malaria [10], it also documents the difficulty in control and elimination of *P. vivax* [18].

### Implications

The WHO certification of malaria elimination requires applicant countries to provide evidence that 1) local malaria transmission has been fully interrupted, resulting in zero indigenous human malaria cases for at least the past three consecutive years (36 months), and 2) an adequate program for preventing reintroduction of malaria transmission is fully functional throughout the country [39, 40]. The last indigenous malaria case in China was reported from Yingjiang County in the Yunnan border area in April 2016 [41]. Malaria elimination in the Yunnan border area has strongly contributed to the remarkable achievement that the WHO certified China malaria-free status on June 30, 2021 [5, 11]. The results of this study helped public health decision-makers planning cost-effective strategies of malaria elimination gearing to the high-risk locations and populations [11]. However, the Yunnan border area is still challenged by reintroduction of malaria transmission. The threat of imported malaria from the border areas of Myanmar will continuously exist for a long time. With understanding the local risk factors of malaria infection, the ‘‘3 + 1’’ strategy for intensive surveillance, rapid response and border collaboration for malaria elimination was developed to reduce the threat of imported malaria for malaria elimination and preventing reintroduction of malaria transmission in the Yunnan border area [11].

Reduced border collaboration of malaria had ever led to slightly resurgent malaria in some border areas of Myanmar since 2014 [11, 15, 18]. The Laiza and nearby areas of the KR2 with a population of approximately 30 thousand persons is one of the malaria hotspot areas along China–Myanmar border due to the risk factors presented in this paper and the weak health services [11]. The number of malaria cases increased from 518 in 2013 to 2,367 in 2016 in the Laiza and nearby areas. The strengthened collaborative interventions between China and Myanmar during 2017–2019 reduced the number of malaria cases to 274 in 2019 [11]. However, reduced collaborative interventions due to the COVID-19 pandemic led to malaria resurgence again. In 2021, a total of 1,532 cases were reported in the Laiza and nearby areas of KR2. This led to importation of most of malaria cases in Yunnan from the Laiza and nearby areas in 2021. Yunnan province mainly borders Myanmar’s five special regions that are out of the central government management of Myanmar Union. The military conflicts and the weak health services cannot effectively control malaria in the five special regions. The risk factors presented in this paper can be referred in planning cross-border collaboration of malaria control.

### Limitations

This study has certain weaknesses, which might be the limitations of the case–control study itself too. 1) To mitigate the confounding effect, controls were matched with age, sex, healthy status, health facilities and treatment-seeking dates. The matching criteria make the study lose the chance to identify whether they are an independent risk or confounding factors of malaria infection. 2) Because the Laiza and nearby areas are the main malaria hot spot that exported most of malaria cases (> 80%) detected in Yunnan, this study only recruited the subjects from the Laiza city hospital in Myanmar. Due to malaria scarceness, the subjects were enrolled from 37 health facilities in China. The large difference in the number of health facilities involved in this study between China and Myanmar may be one of weakness. However, malaria cases that were recruited in China were imported from most parts of the border areas of Myanmar. This would not lead to selection bias. 3) Among the 304 controls recruited in China, 259 (85.2%) had no overnight history in Myanmar in one month prior to attendance of health facilities. The high proportion of non-malaria febrile patients without overnight history in Myanmar might influence assessment of other potential risk factors. However, this can document that overnight in Myanmar is an obvious risk factor for Chinese people. 4) The subjects were only recruited in the health facilities. If some patients just bought anti-malarial drugs from the drug stores for malaria treatment and the self-medication worked, they might not seek diagnosis and treatment in the health facilities. This occasion might lead to exclusion of them from the study. However, with reduction of malaria burden, anti-malarial drugs are disappearing from drug stores because of losing chance of making money from anti-malarial drugs. This leads to anti-malarial drugs mainly available in the public health facilities in China. The chance of the selection bias should be small. 5) Some participants declined to answer certain questions that they thought of as sensitive, this might cause responding bias. 6) To avoid recall bias, most of variables were defined to collect information in one month prior to attendance of health facilities. This may miss to collect enough valuable information. 7) Primaquine is administered over a 14-day period in Myanmar [18] and for double eight days in China [42] to kill liver-stage parasites. With primaquine administration for radical cure treatment, the proportion of *P. vivax* malaria relapse was considered low, so that the previous malaria history was not included in the questionnaires in the study design. The previous malaria history did not thereby included in the LRA model. Any further study should consider the issue of the relapse of *P. vivax* malaria in the study area.

## Conclusion

All malaria cases were infected in Myanmar. The Laiza and nearby areas are the dominant reservoirs of malaria parasites along China–Myanmar border. *Plasmodium vivax* is the dominant malaria parasites, and most malaria patients are male and adult. The independent risk factors of malaria infection are overnight out of home, staying in rural lowland and foothill, low altitude areas and nearby local vector breeding sites in the border areas of Myanmar; and subjects lacking knowledge of malaria transmission. To prevent reintroduction of malaria transmission in China and achieve the WHO malaria elimination goal in the GMS, cross-border collaboration is continuously necessary. Health education is sorely needed for people in China to maintain their malaria knowledge and vigilance, and in Myanmar to promote their ability to apply personal protection.

## Supplementary Information

**Additional file 1.** Additional tables.

## Data Availability

Not applicable.

## Abbreviations

AOR: Adjusted odds ratio
CI: Confidence interval
COVID-19: Coronavirus disease 2019
WHO: World Health Organization
GMS: Greater Mekong Subregion
KR2: Kachin Special Region II
FWI: Family wealth index
LRA: Logistic regression analysis
SR2: Shan Special Region II
